# Supplementary material for: Hidden stories of caregivers with children living with sickle cell disease in Uganda: Experiences, coping strategies and outcomes
Source: PLoS One. 2025 Mar 3;20(3):e0296587. doi: 10.1371/journal.pone.0296587 (PMC11875354; doi:10.1371/journal.pone.0296587)
Supplement: S1 Table — (PDF) [file pone.0296587.s001.pdf]

TABLE SI: PARTICIPANTS DEMOGRAPHIC CHARACTERISTICS

**Participants**	**Age**	**sex**	**marital status**	**birth order**	**no. of children with sickle cell**	**Occupation**	**duration of care**
1	Age 27:	female	married	1st born	1 child	Chef	2 years:
2	Age 32:	female	married	2nd born	1 child	Caretaker:	3 years:
3	Age 33:	male	married	3rd born	1 child	Stay-at-home parent:	8 years:
4	Age 35:	female	married	1st born	1 child	Peasant	13 years
5	Age 36:	female	married	1st born	1 child	Businesswoman:	14 years
6	Age 39:	female	married	2nd born	2 children	Farmer:	17 years
7	Age 40:	female	divorced	1st born	1chid	Teacher:	10 years
8	Age 41:	male	married	1st born	1 child	None	15 years
9	Age 44:	female	married	1st born	1 child	Chef	14 years
10	Age 45:	female	married	2nd born	1 child	peasant	16 years

11	Age 52:	female	widow	1st born	1 child	peasant	20 years
12	Age 38	female	divorced	2nd born	1 child	Farmer:	12 years
