# Supplementary material for: Hidden stories of caregivers with children living with sickle cell disease in Uganda: Experiences, coping strategies and outcomes
Source: PLoS One. 2025 Mar 3;20(3):e0296587. doi: 10.1371/journal.pone.0296587 (PMC11875354; doi:10.1371/journal.pone.0296587)
Supplement: S1 File — (DOCX) [file pone.0296587.s002.docx]

**Conceptual Map: Hidden stories of caregivers with children living with Sickle Cell Disease**

**Definitions:
Experiences**: These are the personal accounts and observations of caregivers regarding their journey of caring for a child with sickle cell disease, including the initial diagnosis, hospital experiences, and daily challenges.
**Coping Strategies:** These are the specific actions, behaviors, or thoughts employed by caregivers to manage the challenges and stresses associated with caring for a child with sickle cell disease. This can include seeking medical advice, adhering to treatment regimens, and seeking social support.
**Coping Outcomes:** These refer to the results or effects of the coping strategies implemented by caregivers. It can include improved health of the child, reduced stress levels, or enhanced quality of life for both the caregiver and the child.

**Table showing Conceptual Map: Hidden stories of caregivers with children living with Sickle Cell Disease**

| **Theme** | **Subtheme** | **Category** | **Codes** |
| --- | --- | --- | --- |
| Experiences | Early Signs | Symptoms Recognition | "*We noticed the child had frequent fevers and pains*."  “*The child had pain episodes and fatigue."* |
|  | Unexplained Illness | Symptoms Recognition | "*The child was often sick, we were unsure of the cause."* |
|  | Doctor Consultation | Hospital Experience | "*We didn’t know what was wrong initially*."  “*We took the child to the hospital for check-up."* |
|  | Diagnosis at Mulago | Doctor Consultation | "*We took the child to Mulago for diagnosis."* |
|  | Hospital Experience | Uncertainty | "*We were unsure of the child's condition at first."* |
|  |  | Duration of Stay | "*We were admitted at Mulago for three months*." |
|  |  | High cost of private hospitals | Private Hospitals |
|  |  | Hospitalization | Multiple admissions at Mulago |
|  |  | Diagnosis Confirmation | "*Doctors confirmed it was sickle cell disease*."  Initial diagnosis in November 2022 |
|  | Symptoms Recognition | Symptoms Manifestation | "*The child had frequent headaches and joint pains*." |
|  |  | Child's symptoms | Recognizing symptoms of SCD |
|  | Challenges with school fees and attendance | Difficulty affording school fees and supplies | Education |
|  | Body-building and blood-enhancing foods |  | Food choices |
|  | Hospital Consultation | Medical Consultation | "*We took the child to the hospital for check-up*." |
|  | **Financial Strain** | High cost of living in Uganda | Financial Burden |
| Coping Strategies | Medication Prescribed | Treatment Routine | "*The doctor prescribed folic acid penicillin*." |
|  |  | Treatment | Use of herbal medicines and painkillers |
|  | Folic Acid Treatment | Treatment Regimen | "*We were given folic acid initially."* |
|  | Research at Makerere | Treatment Continuation | "We participated in a research study at Makerere." |
|  | Family Support | Treatment Compliance | "*Family helped with treatment adherence*."  “*Family helped financially with treatment costs."*  “*Family support helped in continuing the treatment*." |
|  |  | Support from uncle, mother, and grandmother  Neighbors' assistance during sickness | Support  Neighbors support |
|  |  | Proper clothing and mosquito net use  Family support  Nutritious diet including eggs, fish, and vegetables | Clothing  Support from relatives and father  Nutrition |
|  | Social support | Limited interaction with neighbors due to stigma  Cooperation with spouse in caregiving | Stigma  Spousal Cooperation |
|  | Herbal remedies to boost immunity and increase blood count | Local remedies like papaya leaves, avocado leaves, hibiscus, and beetroot  Routine for medication and water intake | Herbal Remedies  Routine, "Overall" Protection |
|  | Continuous pleading to keep the child in school |  | Pleading |
|  | Regular prayer and participation in Anglican church services |  | Prayer |
|  | **Seeking Additional Support** | Requesting leaves from work for caregiving  Advocating for external support for families with sickle cell children | Work Leave  External Support |
|  | Job Flexibility | Work Adjustments | "*My job was flexible."*  *“My workplace was supportive of my situation*." |
| Coping Outcomes | Health Improvements | Adaptation | "*With the medication, the child's condition improved."* |
|  | Health Stabilization | Financial Management | "*The medication helped manage the disease."* |
|  | Doctor Visits | Medical Consultation | *"We took the child to the doctor for check-up."* |
|  | Hospital Stay | Duration of Stay | *"We were admitted at Mulago for three months."* |
|  | Health Maintenance | Employment Stability | "*The medication helped in managing the symptoms."* |
|  | Health Progression | Financial Assistance | "*The treatment helped in stabilizing the condition."* |
|  | Health Improvement | Employment Adaptation | "*The child's condition improved with new medication."* |
|  | **Positive Aspects of Caregiving** | No regrets despite challenges | No regrets |
|  | **Personal Well-being** | Sleep disturbances due to worrying  Mental and emotional stress | Sleep Disturbances  Emotional Stress |
|  | Emotional and physical strain |  | Emotional toll |
|  | Lack of sleep due to worries |  | Sleep deprivation |
|  | Finding happiness in being alive |  | Gratitude |
