# Supplementary material for: Hidden stories of caregivers with children living with sickle cell disease in Uganda: Experiences, coping strategies and outcomes
Source: PLoS One. 2025 Mar 3;20(3):e0296587. doi: 10.1371/journal.pone.0296587 (PMC11875354; doi:10.1371/journal.pone.0296587)
Supplement: Attachment — Submitted filename: response to the reviewers september final.docx [file pone.0296587.s007.docx]

Alinda Isaac	[alindaisaac53@gmail.com](mailto:alindaisaac53@gmail.com)	+256-778-980-221/ +256-757257670	9^th^ September, 2024

Editor-in-Chief
PLOS ONE
Email: plosone@plos.org

**Manuscript ID**: PONE-D-23-37777
**Manuscript Title:** Hidden stories of caregivers with children living with Sickle Cell Disease: experiences, coping strategies and outcomes

Dear Editor-in-Chief and Reviewers,

**RE: Response to Reviewers for Revised Manuscript Submission**
I would like to express my gratitude for the constructive feedback provided by the academic editor and reviewers on my manuscript titled “Hidden stories of caregivers with children living with Sickle Cell Disease: experiences, coping strategies and outcomes”.

I have carefully considered each comment and made revisions to address the concerns raised. Below is a point-by-point table showing the comments, response and the page to the reviewers' comments:

| Comments | Response | Page |
| --- | --- | --- |
| Introduction  1. While the authors have provided some background information on Uganda, they should give some specific details. For instance, how much does the treatment cost? What is the average income of families in Uganda?  Providing this information would aid readers in appreciating the financial challenges families face. | Thank you for your insightful comments and for guiding us toward improving the manuscript.  We appreciate your suggestion to provide more specific details about the financial burden that families face in Uganda regarding sickle cell disease (SCD) treatment.  We hope this added information will help readers better understand the financial challenges faced by families caring for children with SCD. | Page 4 |
| **Methods**  1. Member checking is not a required step for reflexive thematic analysis, and certainly, it is not done to ensure data saturation. Please, revise that statement in the methods section. | Thank you, the reviewer, for your valuable feedback and for bringing this point to our attention.  Upon reviewing our manuscript, we realize that there may have been a misunderstanding regarding member checking. We did not intend to suggest that member checking was employed as part of our reflexive thematic analysis approach, nor did we imply that it was used to ensure data saturation. In fact, we did not conduct member checking as part of the analysis process, as it is not a required step in reflexive thematic analysis.  We have reviewed the methods section and can confirm that no mention of member checking was included. | Page 9 |
| **Results**  1. What is the rationale for summarizing the table showing the characteristics of the participants. There was no comment against the previous version. A reversion to the earlier format is recommended. | Thank you for your valuable feedback regarding the table showing the characteristics of the participants.  I would like to explain that the reason for summarizing the table in the current format was in response to feedback received during the preprint screening process on medRxiv.  The original table, which included more specific participant details, was flagged as potentially identifying information that contravened the platform’s policies on patient confidentiality and anonymity. In particular, the screening process raised concerns about the inclusion of precise ages and other details that could allow participants or their families to be identified. As a result, I was asked to remove or replace such details with broader ranges to protect participant anonymity.  In adherence to those guidelines and to ensure compliance with ethical standards, I summarized the participant characteristics in a manner that balances providing relevant information while maintaining participant confidentiality.  We hope this explanation clarifies the rationale for the current table format. However, if the journal requires a reversion to the earlier version, I am happy to work with the editorial team to find a suitable solution that ensures both compliance and detail.  Thank you again for your understanding and consideration. | Page 11 |
| **Discussion**  1. The authors maintain that reviewing extant literature is helpful to the interpretation of their data. I would be happy to have some citations that support this exercise. | Thank you for your insightful comment regarding the use of extant literature to support the interpretation of our data.  We agree that reviewing existing literature is essential for contextualizing our findings and strengthening the overall interpretation.  In the revised manuscript, we have included additional citations to support our data interpretation.  Below is some of the key sources we referenced to support our interpretation:  1. Hernandez AG, Kiyaga C, Howard TA, Ssewanyana I, Ndeezi G, Aceng JR, et al. Operational analysis of the national sickle cell screening programme in the Republic of Uganda. Afr J Lab Med [Internet]. 2021 Aug 12 [cited 2024 Sep 8];10(1). Available from: <https://ajlmonline.org/index.php/ajlm/article/view/1303>  We are hopeful that this strengthens the manuscript by anchoring our analysis in established literature.  Thank you for your valuable suggestion, and we hope the changes address your concern. | Page 31 |
| 1. I do not see ‘gender imbalance’ as a limitation – many of the caregivers are generally females so what you have is a true reflection of the population. In other words, you don’t have to state fewer male participation as a limitation. | Thank you for your valuable feedback regarding the mention of ‘gender imbalance’ as a limitation.  Upon reflection, I agree with your observation that the higher representation of female caregivers is a true reflection of the population, as women often serve as primary caregivers in many contexts, including in this study.  In light of your suggestion, I have removed the mention of ‘fewer male participation’ as a limitation in the revised manuscript. I appreciate your insight in helping to present the findings more accurately. | Page 26 |
| 1. On page 29, the main author has been described as “DM”. What does that mean? | Thank you for pointing out the discrepancy regarding the author's initials.  This was an oversight, and I have corrected the main author's initials from "DM" to "AI" in the revised manuscript.  I appreciate your careful review and attention to detail. | Page 27 |

Additional Changes Made:

1. I have reviewed the reference list to ensure it is complete and correct. I can confirm that none of the references cited have been retracted. However, in the revised manuscript, I have added one additional reference, bringing the total number of references to 27, compared to the previous 26. All other references remain unchanged and are current and relevant to the study.
2. Financial Disclosure: We acknowledge the financial support granted by the Fogarty International Center of the National Institutes of Health, US, Department of State’s Office of the U.S Global AIDS Coordinator and Health Diplomacy (S/GAC), and the President’s Emergency Plan for AIDS Relief (PEPFAR), HEPI SHSSU (Health Professions Education and Training for Strengthening the Health System and Services in Uganda) under grant No. 1R25TW011213.

Role of Funders:
The funders played an active role in supporting the study by providing financial resources for data collection, stationery procurement, participant facilitation, data analysis, and dissemination of the study findings. However, they had no role in the study design, data analysis and interpretation, decision to publish, or preparation of the manuscript.

I have uploaded the revised manuscript along with this response letter. I believe that the revisions have strengthened the manuscript, addressing the concerns raised by the reviewers and enhancing the overall quality of the work.

Thank you for considering our revised manuscript for publication in PLOS ONE. We look forward to hearing from you soon.

Sincerely,

Alinda Isaac; [alindaisaac53@gmail.com](mailto:alindaisaac53@gmail.com) (Corresponding author)

Lydia Kabiri; [kabdia4@gmail.com](mailto:kabdia4@gmail.com) (Co-author)

Ssebagala Hadad; [hadadissebagala@gmail.com](mailto:hadadissebagala@gmail.com) (Co-author)
