# Supplementary material for: Hidden stories of caregivers with children living with sickle cell disease in Uganda: Experiences, coping strategies and outcomes
Source: PLoS One. 2025 Mar 3;20(3):e0296587. doi: 10.1371/journal.pone.0296587 (PMC11875354; doi:10.1371/journal.pone.0296587)
Supplement: Attachment — Submitted filename: EAB Comments PONE-D-23-37777.docx [file pone.0296587.s003.docx]

**Hidden stories of caregivers with children living with Sickle Cell Disease: experiences, coping strategies and outcomes**

**Reviewer Comments**

**Abstract**

The use of the term ‘diverges’ in the abstract seems inappropriate. Consider using ‘varies’.

**Introduction**

1. The statement ‘A sickle cell disease’ should just start with ‘sickle cell disease’.
2. “Although they had varied topographical distributions, the alpha-thalassemia trait and Glucose-6- Phosphate Dehydrogenase Deficiency (G6PD) were prevalent with sickle cell illness with children aged 0-24 months”.

This statement is unclear and seems inaccurate. Are the authors considering G6PD and alpha-thalassemia as types of sickle cell disease? They are all hemoglobinopathies but not subsets of sickle cell disease.

3. “Taking care of a child with sickle cell disease can be expensive, and many families in Uganda may find it difficult to pay for the required therapies and drugs.”

It will be more appropriate to provide contextual information on SCD and its management in Uganda in the ‘study setting’ section.

4. The introduction section does not give good background information on caregivers of children with SCD in Uganda. It may be argued that this is the gap the study sought to address. However, this could be presented against the backdrop that managing SCD in Uganda comes with peculiar situations that could make caregivers of children with the condition have some ‘hidden’ experiences that need to be explored.

**Methods**

1. Do not use the term ‘children afflicted by sickle cell disease’. Just say ‘Children living with SCD’.
2. The concept of ‘data saturation’ is not as simple as has been presented in the paper. Consider looking at the paper by Brown and Clarke (2021) To saturate or not to saturate? Questioning data saturation as a useful concept for thematic analysis and sample-size rationales. I believe you would find this paper more insightful on reporting on data saturation and sampling decisions in qualitative research.
3. I am not sure if COVID-19 safety measures such as social distancing and consistent face mask use needed to be strictly adhered to in your study in June 2023. This would have been essential in July 2020, or even 2021. I do not see the need for the emphasis.
4. The mention of Brown and Clarke’s method of inductive thematic analysis, published in 2012, has been explained and updated to reflexive thematic analysis in some publications since 2019. They emphasize reflexivity in the analysis process, not merely following the steps they outlined in their earlier publications for data analysis. Consider looking at more recent publications on reflexive thematic analysis to improve the analysis of your data.
5. “Concurrently, relevant literature concerning caregivers' experiences, coping strategies, and coping outcomes was consulted throughout the data analysis. This external knowledge aided in the comprehension and refinement of the emergent themes.”

I do not seem to appreciate the rationale for the above statement in the data analysis process recommended by Braun and Clarke. I expect refinement of the themes to be achieved through thorough engagement with the data, not extant literature. The latter is more in line with grounded theory methods.

6. Has the conceptual mapping that was utilized in explaining ‘the relationships and associations between themes’ been provided anywhere for reference purposes?
7. Did any potential participant refuse participation? If yes, how many and what were their reasons?
8. How was the accuracy of data translation from Luganda to English ensured?

**Results**

1. I do not see the relevance of (n=12), (n=2) in the manuscript. Delete this style of reporting.
2. As requested earlier, a conceptual map of the codes, sub-themes and themes will be useful in explaining the findings of your study. It will be helpful to summarize your themes and sub-themes at the start of the results section, then provide a brief definition of each before you add your various interpretations and participants’ quotes.
3. I do not understand the sub-theme ‘coping strategies at school’. Such a phrase will be more suitable for the children’s experiences, rather than the experiences of caregivers.
4. Again, what is the difference between ‘coping strategies’ and ‘coping outcomes’? A clear definition of each theme as well as the conceptual map of themes and their relationships will help explain this.
5. You mentioned that you used pseudonyms but I only saw numbering as the participant identifiers. Please, revisit and address that.

**Discussion**

1. The discussion section has been done satisfactorily.
2. ‘Hidden’ or ‘concealed’ are not appropriate terms to use to describe the experiences of caregivers – consider using ‘untold’.
3. In the ‘strengths and limitations of the study’ section, generalizability is mentioned as a potential limitation. This is inaccurate and not a limitation for any qualitative study – transferability has always been the goal.
4. I do not see the ‘gender imbalance’ as a problem when it reflects the picture of caregivers who are involved in the care of children with SCD.

Authors’ contributions – what was the role of SH, apart from reading the final version of the manuscript?

The work will benefit from major proofreading as the errors are quite too many.
