# Supplementary material for: Hidden stories of caregivers with children living with sickle cell disease in Uganda: Experiences, coping strategies and outcomes
Source: PLoS One. 2025 Mar 3;20(3):e0296587. doi: 10.1371/journal.pone.0296587 (PMC11875354; doi:10.1371/journal.pone.0296587)
Supplement: Attachment — Submitted filename: Response to Reviewers.docx [file pone.0296587.s006.docx]

Alinda Isaac	[alinda53isaac@gmail.com](mailto:isaacalinda53@gmail.com)	+256-778-980-221/ +256-757257670	25^th^ April, 2024

Editor-in-Chief
PLOS ONE
Email: plosone@plos.org

**Manuscript ID**: PONE-D-23-37777
**Manuscript Title:** Hidden stories of caregivers with children living with Sickle Cell Disease: experiences, coping strategies and outcomes

Dear Editor-in-Chief and Reviewers,

**RE: Response to Reviewers for Revised Manuscript Submission**
I would like to express my gratitude for the constructive feedback provided by the academic editor and reviewers on my manuscript titled “Hidden stories of caregivers with children living with Sickle Cell Disease: experiences, coping strategies and outcomes”. I have carefully considered each comment and made revisions to address the concerns raised. Below is a point-by-point table showing the comments, response and the page to the reviewers' comments:

| Comments | Response | Page |
| --- | --- | --- |
| **Abstract**  The use of the term ‘diverges’ in the abstract seems inappropriate. Consider using ‘varies’. | I want to the thank the reviewer for this suggestion.  I have really changed it | Page 2 |
| Introduction   1. The statement ‘A sickle cell disease’ should just start with ‘sickle cell disease’. | Thank you, the reviewer, for the grammatical correction.  I have corrected it. | Page 3 |
| 2. “Although they had varied topographical distributions, the alpha-thalassemia trait and Glucose-6- Phosphate Dehydrogenase Deficiency (G6PD) were prevalent with sickle cell illness with children aged 0-24 months”.   This statement is unclear and seems inaccurate. Are the authors considering G6PD and alpha-thalassemia as types of sickle cell disease? They are all hemoglobinopathies but not subsets of sickle cell disease. | Thank you, the reviewer, for bringing to our attention the clarity and accuracy concerns regarding the statement on the prevalence of alpha-thalassemia trait and Glucose-6-Phosphate Dehydrogenase Deficiency (G6PD) in children with sickle cell illness.  You are correct in noting that alpha-thalassemia trait and G6PD deficiency are not subsets of sickle cell disease but rather distinct hemoglobinopathies. The intent of our statement was to highlight their co-occurrence with sickle cell disease in the studied population.  We acknowledge that the wording may have caused confusion. | Page 4 |
| 3. Taking care of a child with sickle cell disease can be expensive, and many families in Uganda may find it difficult to pay for the required therapies and drugs.”   It will be more appropriate to provide contextual information on SCD and its management in Uganda in the ‘study setting’ section. | Thank you for your feedback regarding the 'study setting' section of our manuscript. You pointed out the importance of providing contextual information on the economic challenges associated with managing SCD in Uganda.  We appreciate this suggestion as it indeed offers a more comprehensive understanding of the environment in which our study was conducted. | Page 6 |
| 4. The introduction section does not give good background information on caregivers of children with SCD in Uganda. It may be argued that this is the gap the study sought to address. However, this could be presented against the backdrop that managing SCD in Uganda comes with peculiar situations that could make caregivers of children with the condition have some ‘hidden’ experiences that need to be explored. | Thank you, the reviewer for insightful suggestion  I have rectified the issue | Page 5 |
| **Methods**   1. Do not use the term ‘children afflicted by sickle cell disease’. Just say ‘Children living with SCD’. | Thank you, the reviewer for this suggestion, I have accepted to change the word. | Page 6 |
| 2. The concept of ‘data saturation’ is not as simple as has been presented in the paper. Consider looking at the paper by Brown and Clarke (2021) To saturate or not to saturate? Questioning data saturation as a useful concept for thematic analysis and sample-size rationales. I believe you would find this paper more insightful on reporting on data saturation and sampling decisions in qualitative research. | Thank you for pointing out the complexity and nuances associated with the concept of 'data saturation' in qualitative research.  I appreciate your recommendation to consult the paper by Brown and Clarke (2021) for a more in-depth understanding of this topic. In our study, we aimed to achieve data saturation, but I acknowledge that our initial presentation of this concept may have been oversimplified.  To provide a clearer picture of our approach to data saturation and sampling decisions, I revisited the relevant literature and incorporate a more detailed explanation in the revised manuscript.  This includes a discussion of how we determined sample size, monitored data saturation throughout the data collection process, and made informed decisions regarding the adequacy of our sample size for achieving saturation. | Page 7 to Page 8 |
| 3. I am not sure if COVID-19 safety measures such as social distancing and consistent face mask use needed to be strictly adhered to in your study in June 2023. This would have been essential in July 2020, or even 2021. I do not see the need for the emphasis. | Thank you for your feedback regarding the inclusion of COVID-19 safety measures in our study conducted in June 2023.  While it may seem that these precautions were less necessary by that time, we prioritized the safety and well-being of our participants and their caregiving roles.  Additionally, adherence to COVID-19 restrictions was a requirement stipulated in the ethical approval granted by our institution, further underscoring the importance of these measures.  Given the ongoing uncertainty surrounding vaccination status and the potential need for booster doses in our study population, we deemed it essential to adhere to standard and transmission-based precautions to minimize any potential harm or risk of transmission during data collection.  We appreciate your understanding of our commitment to ensuring the safety of our participants. | Page 8 |
| 4. The mention of Brown and Clarke’s method of inductive thematic analysis, published in 2012, has been explained and updated to reflexive thematic analysis in some publications since 2019. They emphasize reflexivity in the analysis process, not merely following the steps they outlined in their earlier publications for data analysis. Consider looking at more recent publications on reflexive thematic analysis to improve the analysis of your data. | Thank you for pointing out the evolution of Brown and Clarke's method to reflexive thematic analysis in more recent publications. We acknowledge the importance of reflexivity in the analysis process.  In our study, we did incorporate reflexivity by continuously reflecting on our interpretations and discussing them within the research team to ensure they were grounded in the data and not influenced by our preconceptions.  While we referenced Brown and Clarke's method to provide a framework and elaboration on how the analysis was conducted, we recognize that the field has evolved, and we appreciate your suggestion to look into more recent publications on reflexive thematic analysis. | Page 9 |
| 5. Concurrently, relevant literature concerning caregivers' experiences, coping strategies, and coping outcomes was consulted throughout the data analysis. This external knowledge aided in the comprehension and refinement of the emergent themes.”   I do not seem to appreciate the rationale for the above statement in the data analysis process recommended by Braun and Clarke. I expect refinement of the themes to be achieved through thorough engagement with the data, not extant literature. The latter is more in line with grounded theory methods. | I appreciate the reviewer for the insights and I would like to clarify the rationale behind consulting relevant literature during our data analysis.  While we acknowledge the importance of the grounded theory approach, which emphasizes the generation of themes directly from the data, we also believe that incorporating existing literature can provide additional context and depth to our findings. The literature serves as a reference point that can help to validate, challenge, or expand upon the emergent themes from our data.  In our study, which focuses on the experiences, coping strategies, and coping outcomes of caregivers of children with sickle cell disease, the extant literature provided valuable insights into the broader context of caregiving, similar experiences in other chronic illnesses, and evidence-based coping strategies.  This external knowledge aided us in understanding the complexities of the caregivers' experiences and in refining our emergent themes to ensure they were both grounded in the data and reflective of the broader literature. | Page 10 |
| 6. Did any potential participant refuse participation? If yes, how many and what were their reasons? | Thank you, the reviewer, for the valuable insight.  However, none of the potential participants declined to participate in our study. All caregivers approached consented willingly to take part, and there was no coercion involved.  We explained the purpose and details of the study to them both before obtaining their consent and again during the data collection process to ensure they fully understood and felt comfortable sharing their experiences. | Page 7 |
| 7. How was the accuracy of data translation from Luganda to English ensured? | Thank you for raising a valuable point regarding the accuracy of data translation from Luganda to English  To ensure the fidelity and accuracy of the translation process, the following steps were undertaken:  Qualified Translators: The audio recordings were transcribed and translated by qualified and experienced translators proficient in both Luganda and English languages.  Supervision by Principal Investigator: The entire translation process was conducted under the close supervision of the principal investigator, A.I., who is fluent in both languages and reviewed the translated transcripts for accuracy and contextual relevance.  Consultation with Participants: Where necessary, the translated transcripts were reviewed with participants to ensure that the translated content accurately represented their original statements and meanings. | Page 9 |
| **Results**   1. I do not see the relevance of (n=12), (n=2) in the manuscript. Delete this style of reporting. | Thank you, the reviewer for the suggestion, I have deleted this style of reporting | Page 11 |
| 2. As requested earlier, a conceptual map of the codes, sub-themes and themes will be useful in explaining the findings of your study. It will be helpful to summarize your themes and sub-themes at the start of the results section, then provide a brief definition of each before you add your various interpretations and participants’ quotes. | Thank you, the reviewer for the suggestion,  I have attached a separate file for the conceptual map to this manuscript |  |
| 3. I do not understand the sub-theme ‘coping strategies at school’. Such a phrase will be more suitable for the children’s experiences, rather than the experiences of caregivers. | Thank you for your feedback regarding the sub-theme  The sub-theme aimed to capture the unique experiences, challenges, and coping strategies employed by caregivers in navigating the educational system and supporting their children's academic and social needs.  We believe that understanding these strategies is crucial for gaining insights into the holistic care and support provided by caregivers to children with SCD. | Page 18 |
| 4. You mentioned that you used pseudonyms but I only saw numbering as the participant identifiers. Please, revisit and address that. | Thank you for pointing out the discrepancy regarding the use of pseudonyms for participant identifiers in our manuscript  We appreciate your careful review and we have clarified this in aspect of our study. | Page 9 |
| **Discussion**   1. The discussion section has been done satisfactorily. 2. ‘Hidden’ or ‘concealed’ are not appropriate terms to use to describe the experiences of caregivers – consider using ‘untold’. | Thank you for your valuable feedback,  I understand your perspective, but our choice of the term 'hidden' was deliberate and reflective of the study's focus. Our intention was to emphasize that caregivers often hold back or do not openly share certain aspects of their experiences, which we aimed to uncover through our research  I recognize that language is crucial in accurately conveying the essence of our findings. To address your concern, we will consider the use of 'untold' and revise the manuscript accordingly if it does not compromise the original intent and message of our study. | Page 28 |
| 3. In the ‘strengths and limitations of the study’ section, generalizability is mentioned as a potential limitation. This is inaccurate and not a limitation for any qualitative study – transferability has always been the goal. | Thank you for pointing out the distinction between generalizability and transferability in qualitative research  We appreciate your feedback and agree that transferability is indeed the appropriate term to use when discussing the applicability of our study findings to other contexts or settings. | Page 27 |
| 4. I do not see the ‘gender imbalance’ as a problem when it reflects the picture of caregivers who are involved in the care of children with SCD. | Thank you for your insightful comments regarding the gender imbalance observed in our study  In response to your feedback, we have revised the 'Strengths and Limitations of the Study' section to better address this issue: | Page 27 |
| Authors’ contributions – what was the role of SH, apart from reading the final version of the manuscript? | Thank you, the reviewer for this observation, I have adjusted on the role that S.H played | Page 30 |
| The work will benefit from major proofreading as the errors are quite too many. | Thank you for your valuable feedback regarding the proofreading of our manuscript.  We sincerely apologize for the oversights and errors that were present in the initial submission. We have now conducted a thorough proofreading and editing process to rectify these issues. |  |

Additional Changes Made:

1. Referencing the table Ⅰ, used in the manuscript
2. Financial Disclosure: We acknowledge the financial support granted by the Fogarty International Center of the National Institutes of Health, US, Department of State’s Office of the U.S Global AIDS Coordinator and Health Diplomacy (S/GAC), and the President’s Emergency Plan for AIDS Relief (PEPFAR), HEPI SHSSU (Health Professions Education and Training for Strengthening the Health System and Services in Uganda) under grant No. 1R25TW011213.

Role of Funders:
The funders played an active role in supporting the study by providing financial resources for data collection, stationery procurement, participant facilitation, data analysis, and dissemination of the study findings. However, they had no role in the study design, data analysis and interpretation, decision to publish, or preparation of the manuscript.

I have uploaded the revised manuscript along with this response letter. I believe that the revisions have strengthened the manuscript, addressing the concerns raised by the reviewers and enhancing the overall quality of the work.

Thank you for considering our revised manuscript for publication in PLOS ONE. We look forward to hearing from you soon.

Sincerely,

Alinda Isaac; [alindaisaac53@gmail.com](mailto:alindaisaac53@gmail.com) (Corresponding author)

Lydia Kabiri; [kabdia4@gmail.com](mailto:kabdia4@gmail.com) (Co-author)

Ssebagala Hadad; [hadadissebagala@gmail.com](mailto:hadadissebagala@gmail.com) (Co-author)
