# Supplementary material for: Hidden stories of caregivers with children living with sickle cell disease in Uganda: Experiences, coping strategies and outcomes
Source: PLoS One. 2025 Mar 3;20(3):e0296587. doi: 10.1371/journal.pone.0296587 (PMC11875354; doi:10.1371/journal.pone.0296587)
Supplement: Attachment — Submitted filename: EAB Comments PONE-D-23-37777 R1.docx [file pone.0296587.s005.docx]

I am pleased that the authors have made some changes and/or provided further explanation to some sections of the manuscript. These have made the manuscript more insightful. There are a few more issues that need further attention, though.

1. While the authors have provided some background information on Uganda, they should give some specific details. For instance, how much does the treatment cost? What is the average income of families in Uganda?

Providing this information would aid readers in appreciating the financial challenges families face.

2. Member checking is not a required step for reflexive thematic analysis, and certainly, it is not done to ensure data saturation. Please, revise that statement in the methods section.

3. What is the rationale for summarizing the table showing the characteristics of the participants. There was no comment against the previous version. A reversion to the earlier format is recommended.

4. The authors maintain that reviewing extant literature is helpful to the interpretation of their data. I would be happy to have some citations that support this exercise.

5. I do not see ‘gender imbalance’ as a limitation – many of the caregivers are generally females so what you have is a true reflection of the population. In other words, you don’t have to state fewer male participation as a limitation.

6. On page 29, the main author has been described as “DM”. What does that mean?
